# The impact on patient health and service outcomes of introducing nurse consultants: a historically matched controlled study

**DOI:** 10.1186/1472-6963-13-431

**Published:** 2013-10-23

**Authors:** Diana TF Lee, Kai Chow Choi, Carmen WH Chan, Sek Ying Chair, Dominic Chan, Sylvia YK Fung, Eric LS Chan

**Affiliations:** 1 Chair Professor of Nursing and Director, The Nethersole School of Nursing, The Chinese University of Hong Kong, Hong Kong, SAR, China; 2 The Nethersole School of Nursing, 7/F, Esther Lee Building, The Chinese University of Hong Kong, Shatin, Hong Kong, SAR, China; 3 Professor, The Nethersole School of Nursing, The Chinese University of Hong Kong, Hong Kong, SAR, China; 4 Formerly Associate Professor, The Nethersole School of Nursing, The Chinese University of Hong Kong, Hong Kong, SAR, China; 5 Formerly Nursing Director, Hospital Authority, Hong Kong, SAR, China; 6 Principal Nursing Officer, Hospital Authority, Hong Kong, SAR, China

**Keywords:** Nurse consultant, Patient outcomes, Patient satisfaction, Hong Kong

## Abstract

**Background:**

The position of nurse consultant (NC) was introduced in Hong Kong by the Hospital Authority in January 2009. Seven NCs were appointed in five clinical specialties: diabetes, renal, wound and stoma care, psychiatrics, and continence. This was a pilot to explore the impact of the introduction of NCs on patient health and service outcomes.

**Methods:**

The present paper describes a historically matched controlled study. A total of 280 patients, 140 in each cohort under NC or non-NC care, participated in the study. The patient health and service outcomes of both cohorts were evaluated and compared: accident and emergency visits, hospital admissions, length of hospital stays, number of acute complications, number of times of treatment or regimen altered by nurses according to patient’s condition, glycated haemoglobin A1c (HbA1c) levels, urea and urea-to-creatinine ratios, and number of wound dressings for patients in corresponding specialty units. A patient satisfaction instrument was also used to assess the NC cohort.

**Results:**

The study showed that patients under NC care had favourable patient health and service outcomes compared with those under non-NC care. The NC cohort also reported a high level of patient satisfaction.

**Conclusions:**

The study demonstrates that the introduction of NCs in specialty units may have a positive impact on patients’ health and service outcomes. The high level of patient satisfaction scores indicates that patients appreciate the care they are receiving with the introduction of NCs.

## Background

In January 2009, a new position, nurse consultant (NC), was introduced into the new Nurses Career Structure and Progression Model in Hong Kong by the Hospital Authority, which manages all the public hospitals and covers about 80% of in-patient hospital services in Hong Kong [1]. The primary aim of introducing of NCs was to broaden the nursing clinical career path. Under the new structure, a registered nurse can progress to advanced practice nurse (APN) and NC in a specific clinical field. The first batch of seven NCs in Hong Kong were appointed in January 2009 in five clinical specialties: diabetes, renal, wound and stoma care, psychiatrics, and continence. The new NCs were appointed to seven different specialty units in Hong Kong public hospitals.

In Hong Kong, there are suggested requirements of an NC in four main areas: academic, research, clinical and leadership competency. An NC is required to have at least a Master degree, preferably doctoral, in clinical streams of nursing, completed nursing specialty training, preferably with general training in other specialties, and track record of demonstrated specialty educational planning and teaching experiences. Experiences on knowledge development, implementation and transfer are also expected from an NC. Regarding clinical competency, an NC is anticipated to have at least eight years of specialty clinical experience, preferably in advanced roles and a track record of demonstrated specialty expert clinical skills and care innovations. Furthermore, demonstrated leadership and management skills with cross-specialty and inter-professional working experiences are also desirable for an NC.

The NC position was first established in healthcare settings more than a decade ago in the United Kingdom [2]. It may even be dated back to 1986 in New South Wales, Australia [3], while different advanced nursing practice titles, such as 'nurse specialist’ or 'nurse practitioner’ , bearing a certain similarity in role and scope of practice to NCs have developed in many countries in the last few decades [4]. Indeed, in some states of Australia and the United States, a nurse specialist may actually fulfill an NC role [5,6]. Although these various advanced nursing practice positions share a degree of similarity in their roles and scope of practice, the NC is expected to achieve better outcomes for patients by further enhancing the quality of care processes and services provision, and providing career development opportunities for experienced nurses to remain in clinical practice [2].

A growing body of literature is helping to clarify the scope and boundaries of the NC’s role. In particular, some officially recognised work indicates that the role of the NC is essentially based on clinical practice, with expert functions in four essential domains: practice and service development, leadership and consultancy, education and training, and research and evaluation [7-9]. However, limited research-based studies have been published to evaluate the impact of NCs on healthcare quality [10-12]. Kennedy et al. [13] suggest that future research should be conducted to evaluate the impact of NC care on patient outcomes and experiences. The present study focuses on the patient health and service outcomes which are expected to be sensitive to the practice and service development as well as to the educational role of NCs.

We have conducted a pilot study to review and evaluate, in a preliminary and independent way, the first wave of new NC positions in Hong Kong public hospitals, using a mixed method design with both qualitative and quantitative approaches [14]. The present study is a sub-study, using only quantitative approach, to explore the impact on selected patient health and service outcomes of the introduction of NCs to Hong Kong public hospitals.

## Methods

This was a historically matched controlled study and, as it was a pilot to explore the effect of introducing NCs on major patient health and service outcomes, no prior sample size calculation was undertaken. The sample size was not determined upon hypothesis testing but was anticipated to provide adequate information about the feasibility of evaluating the impact of NCs on patient outcomes, particularly assessing the logistics of subject recruitment and data collection, the feasibility of selecting matched controls, and estimating the effect sizes on the outcome measures.

The records of patients from April to June 2008 in the seven specialty units with NCs deployed after January 2009 were obtained from the Clinical Management System (CMS), Hong Kong’s publicly funded government hospitals system. The CMS has been operating since 1996, and records all patient data in each episode of in- or out-patient care in all institutions, including all public hospitals, which are linked with it.

Eligible NC-care patients were those Hong Kong residents who aged 18 years or above and consented to participate in the study. The first 20 eligible patients admitted after 1 April 2009 (the indexed hospital admission date of the NC care cohort) to each of the seven units were selected for the study. For each patient selected in 2009 (exposed patient), a patient matched for disease type (based on primary diagnosis with the same ICD code), sex and age within 5 years of interval (control patient) was retrospectively selected from the same unit with the closest hospital admission date in 2008 (the indexed hospital admission in the non-NC care cohort) to that of the exposed patient. An ideal pair would be exposed and control patients admitted on the same date but one year apart, in 2009 and 2008 respectively, to minimize the seasonal effect. The exposed and control patients constituted the NC care and non-NC care cohorts, respectively, for the patient outcome evaluation study. The participating patients’ CMS records for the first six months after their indexed hospital admission were also retrieved for evaluation.

The information collected includes demographic characteristics (NC cohort only), patient health and service outcomes, and patient satisfaction measures (NC cohort only). Patient health and service outcomes were selected on the basis of appropriateness and feasibility [15]. All these outcomes were retrieved from the CMS.

### Patient health and service outcome measures

(1) Accident and emergency visits: total number of accident and emergency visits to any institutions linked with the CMS in the first six months after discharge from the indexed hospital admission.

(2) Hospital admissions and length of hospital stay: total number of hospital admissions and the total length of the hospital stays, in days, during the first six months after discharge from the indexed hospital admission.

(3) Number of acute complications: total number of acute complications defined by the physicians in charge and recorded in the CMS during the hospital stay of the indexed admission.

(4) Number of times of treatment or medication regimen altered by nurses: total occasions when patients’ treatment or medication regimen was altered by nurses because of the patients’ condition and was recorded in the CMS during the indexed hospital stay.

(5) Glycated haemoglobin A1c (HbA1c): the latest HbA1c values for patients admitted to diabetes specialty units taken at the indexed hospital admission and 6 months later and were recorded in the CMS were taken as the pre- and post- HbA1c levels.

(6) Urea level and urea-to-creatinine ratio: the last measurements of urea level and urea-to-creatinine ratio of patients admitted to renal specialty units during the hospital stay of the indexed admission, as recorded in the CMS.

(7) Number of dressings: the total number of dressings for healing wounds of patients admitted to the surgery specialty unit during the hospital stay of the indexed admission, as recorded in the CMS.

### Patient satisfaction

The Chinese version of the patient satisfaction instrument (PSI) [16,17] was used to assess patients’ satisfaction during the indexed hospital stay of the NC cohort. This is a validated 25-item questionnaire with three subscales (technical-professional care, trust and patient education) and a total score quantifying overall patient satisfaction. High sub-scale and total scores indicate greater satisfaction. The Chinese version of PSI demonstrates good reliability with a Cronbach’s alpha of at least 0.85 [17]. Since there are no benchmark values of PSI scores, an empirical approach has been adopted to generate reference ranges for the mean PSI sub-scale and overall scores. All studies published in English during the period 1 August 2003 to 31 July 2013 and reporting PSI scores were extracted through Google Scholar. A total of seven studies with 14 cohorts’ PSI scores reported were identified. (See reference list [18-24]).

### Data analysis

Data were summarized and presented using appropriate descriptive statistics. Normal-like distributed and skewed continuous variables are presented, respectively, by their means (standard deviations) and medians (ranges), unless specified otherwise. Categorical variables and those continuous variables with only a few discrete values are presented by frequency and percentage. Owing to the retrospective nature of the non-NC cohort, no demographic variables except age and gender were collected from that quarter, and only unadjusted comparisons on patient outcomes were made between NC and non-NC cohorts matched by age, sex and type of disease. Patient health and service outcome measures, except length of hospital stay among those admitted to hospital in the first six months after the indexed hospital admission, of the two matched cohorts were compared using Wilicoxon signed-rank test or paired t-test, depending on the underlying distributions of the outcomes [25]. The subgroup analysis of the length of hospital stay for those who had re-admitted to hospital in the first six months after the indexed hospital admission between the two cohorts was compared by Mann–Whitney test. All the statistical analyses were performed using SPSS 18.0 (SPSS Inc, Chicago, IL). All statistical tests were two-tailed and a p-value < 0.05 was considered statistically significant.

### Ethical considerations

Ethical approval was obtained from the ethics committee affiliated with the Hospital Authority of Hong Kong prior to data collection, and written consents were also obtained from the NC cohort. Confidentiality and anonymity of all information was assured, and the right to refuse to participate or withdraw from the study at any time was emphasized.

## Results

A total of 280 patients with 20 age-, sex- and disease type-matched pairs in each of the NC and non-NC care cohorts from each of the seven specialty units were selected for the present patient outcome evaluation study, with 40 pairs recruited from each of the diabetes and renal specialties. The remaining 60 pairs of patients came from psychiatrics, continence, and wound and stoma care specialties, 20 pairs from each. The 140 pairs of patients in the NC and non-NC care cohorts were both composed of 73 (52%) females and 67 males (48%) with a mean age of 55 ± 16 and 59 ± 17 years respectively. The detailed characteristics of the NC care cohort are given in Table 1.

**Table 1:** Demographic characteristics of the NC cohort (n=140)

**Characteristics**	
Age (years) ^ψ^	55.0 (15.7)
Gender	
Female	73 (52.1%)
Male	67 (47.9%)
Educational level	
No formal education	10 (7.2%)
Primary to junior school	78 (56.1%)
High school	43 (30.9%)
College or above	8 (5.8%)
Marital status	
Married	88 (62.9%)
Single	27 (19.3%)
Divorced	10 (7.1%)
Widowed	10 (7.1%)
Declined to disclose	5 (3.6%)
Employment status	
Full/part-time working	42 (30.7%)
Unemployed	21 (15.3%)
Home-maker	38 (27.7%)
Retired	36 (26.3%)
Monthly household income (HK$)	
<10,000	60 (42.9%)
10,000 – 19,999	31 (22.1%)
20,000 – 29,999	14 (10.0%)
≥ 30,000	7 (5.0%)
Declined to disclose	28 (20.0%)
Duration of illness (years)^†, ξ^	5 (1 – 12)
Duration of receiving specialty treatment (weeks)^†, ξ^	35 (9 – 260)

Variables marked with ^ψ^ are presented as mean (standard deviation) and ^†^ are presented as median (interquartile range), all others are frequency (percentage).

^ξ^ Those patients who were in the wound and stoma care specialty units were not counted.

Table 2 shows the availability of the patient health and service outcomes chosen for evaluation in each of the seven specialty units in the analysis. A comparison of patient outcomes between the NC and non-NC care cohorts appears in Table 3. The NC care cohort had a significantly larger proportion of patients without further accident and emergency visits or hospital admissions in the first six months after discharged from their indexed hospital admissions (both p<0.001, Table 3). Furthermore, among those re-admitted to hospitals in the six months, the NC cohort had a significantly shorter length of hospital stay than the non-NC cohort (median = 2 vs 15 days, p=0.001, Table 3). Stratified by specialty, noticeable differences in the above three patient service outcomes were only observed in the diabetes, renal and psychiatrics specialties (Table 3). In the number of acute complications during the indexed hospital stay, there was generally no significant difference found between the two cohorts, except that more patients in the renal specialty of the NC cohort had significantly fewer such complications than in the non-NC cohort (p<0.001, Table 3). The frequency of treatment or medication regimen altered by nurses according to patients’ condition was found to be significantly higher in both diabetes and renal specialties of the NC cohort than in those of the non-NC cohort (Table 3).

**Table 2:** The availability of the patient health and service outcomes for evaluation in each of the seven specialty units included in the analysis

			**Specialty unit**				
**Patient health and service outcomes**	**Diabetes A (n=40)**	**Diabetes B (n=40)**	**Renal A (n=40)**	**Renal B (n=40)**	**Wound & stoma care (n=40)**	**Psychiatrics (n=40)**	**Continence (n=40)**
**Service outcomes**							
Number of Accident & Emergency visits	√	√	√	√	√	√	√
Number of hospital admissions	√	√	√	√	√	√	√
Length of hospital stay	√	√	√	√	√	√	√
Number of acute complications	√	√	√	√	–	–	√
Number of times of treatment / medication regimen altered by nurses according to patients’ condition	√	√	√	√	–	–	√
**Health outcomes**							
HbA1c level	√	√	–	–	–	–	–
Urea level	–	–	√	√	–	–	–
Urea creatinine ratio	–	–	√	√	–	–	–
Number of dressings for wound healed	–	–	–	–	√	–	–

–: not available.

**Table 3:** Patient health and service outcomes between the NC and non-NC care cohorts

	**NC care**				**Non- NC care**				**p-value#**
**Service outcomes**									
Number of Accident & Emergency visits in the first six months after the indexed hospital admission*	0	1	2	≥ 3	0	1	2	≥ 3	
All specialties	133 (95.0%)	6 (4.3%)	1 (0.7%)	0	107 (76.4%)	20 (14.3%)	10 (7.1%)	3 (2.1%)	<0.001
Diabetes	39 (97.5%)	1 (2.5%)	0	0	34 (85.0%)	4 (10.0%)	2 (5.0%)	0	0.053
Renal	37 (92.5%)	3 (7.5%)	0	0	18 (45.0%)	13 (32.5%)	8 (20.0%)	1 (2.5%)	<0.001
Wound & stoma care	20 (100.0%)	0	0	0	20 (100.0%)	0	0	0	–
Psychiatrics	19 (95.0%)	1 (5.0%)	0	0	16 (80.0%)	3 (15.0%)	0	1 (5.0%)	0.157
Continence	18 (90.0%)	1 (5.0%)	1 (5.0%)	0	19 (95.0%)	0	0	1 (5.0%)	0.999
Number of hospital admissions in the first six months after the indexed hospital admission*	0	1	2	≥ 3	0	1	2	≥ 3	
All specialties	133 (95.0%)	7 (5.0%)	0	0	100 (71.4%)	27 (19.3%)	11 (7.9%)	2 (1.4%)	<0.001
Diabetes	38 (95.0%)	2 (5.0%)	0	0	31 (77.5%)	7 (17.5%)	2 (5.0%)	0	0.029
Renal	37 (92.5%)	3 (7.5%)	0	0	18 (45.0%)	13 (32.5%)	8 (20.0%)	1 (2.5%)	<0.001
Wound & stoma care	20 (100.0%)	0	0	0	20 (100.0%)	0	0	0	–
Psychiatrics	19 (95.0%)	1 (5.0%)	0	0	13 (65.0%)	5 (25.0%)	1 (5.0%)	1 (5.0%)	0.034
Continence	19 (95.0%)	1 (5.0%)	0	0	18 (90.0%)	2 (10.0%)	0	0	0.317
*Length of hospital stay among those who had re-admitted to hospital in the first six months after the indexed hospital admission (days)*^*ψ*^	Median (range)				Median (range)				
All specialties	2 (1 – 17)				15 (1 – 88)				0.001^a^
Diabetes	2 (1 – 2)				6 (2 – 17)				0.017^a^
Renal	1 ( 1 – 7)				16 (4 – 88)				<0.001^a^
Wound & stoma care	–				–				–
Psychiatrics	17 (17 – 17)				41 (9 – 72)				0.091^a^
Continence	2 (2 – 2)				2 (1 – 2)				0.799^a^
Number of acute complications during the hospital stay of the indexed admission*	0	1	2	≥ 3	0	1	2	≥ 3	
All specialties	81 (81.0%)	9 (9.0%)	7 (7.0%)	3 (3.0%)	64 (64.0%)	24 (24.0%)	9 (9.0%)	3 (3.0%)	0.112
Diabetes	33 (82.5%)	5 (12.5%)	2 (5.0%)	0	33 (82.5%)	6 (15.0%)	1 (2.5%)	0	0.813
Renal	37 (92.5%)	3 (7.5%)	0	0	15 (37.5%)	15 (37.5%)	8 (20.0%)	2 (5.0%)	<0.001
Wound & stoma care	–	–	–	–	–	–	–	–	–
Psychiatrics	–	–	–	–	–	–	–	–	–
Continence	11 (55.0%)	1 (5.0%)	5 (25.0%)	3 (15.0%)	16 (80.0%)	3 (15.0%)	0	1 (5.0%)	0.058
Number of times of treatment / medication regimen altered by nurses according to patients’ condition during the hospital stay of the indexed admission*	0	1	2	≥ 3	0	1	2	≥ 3	
All specialties	24 (24.0%)	36 (36.0%)	27 (27.0%)	13 (13.0%)	66 (66.0%)	19 (19.0%)	11 (11.0%)	4 (4.0%)	<0.001
Diabetes	6 (15.0%)	21 (52.5%)	12 (30.0%)	1 (2.5%)	23 (57.5%)	6 (15.0%)	9 (22.5%)	2 (5.0%)	0.004
Renal	9 (22.5%)	10 (25.0%)	11 (27.5%)	10 (25.0%)	36 (90.0%)	4 (10.0%)	0	0	<0.001
Wound & stoma care	–	–	–	–	–	–	–	–	–
Psychiatrics	–	–	–	–	–	–	–	–	–
Continence	9 (45.0%)	5 (25.0%)	4 (20.0%)	2 (10.0%)	7 (35.0%)	9 (45.0%)	2 (10.0%)	2 (10.0%)	0.861
**Health outcomes**									
*Diabetes specialty*	Mean (standard deviation)				Mean (standard deviation)				
HbA1c level (%) [pre] ^†^	10.0 (1.9)				9.4 (2.2)				0.156^b^
Hba1c level (%) [post] ^†^	8.2 (1.4)				9.2 (1.8)				0.006^b^
Change of HbA1c level (%) [post – pre] ^†^	-1.8 (1.7)				-0.2 (1.9)				<0.001^b^
*Renal specialty*	Mean (standard deviation)				Mean (standard deviation)				
Urea level (mmol/L) ^†^	21.7 (8.1)				26.2 (10.4)				0.034^b^
Urea creatinine ratio ^†^	27.0 (10.5)				34.8 (12.4)				0.001^b^
Wound & stoma care *specialty*	Median (range)				Median (range)				
Number of dressings for wound healed ^ψ^	7 (1 – 26)				28 (12 – 77)				<0.001

Data marked with ^†^ are presented as mean (standard deviation) and with ^ψ^ as median (interquartile range), all others are presented as frequency (%). *These continuous variables with only a few discrete values are presented by frequency and percentage instead of median (range) or mean (standard deviation).

^#^ All the comparisons were assessed using Wilicoxon signed-rank test, unless specified otherwise.

^a^ Mann–Whitney test for the subgroup analysis of the length of hospital stay for those who had re-admitted to hospital in the first six months after the indexed hospital admission between the two cohorts (totally 7 and 40 patients respectively among the 140 patients in each of the NC and non-NC care cohorts).

^b^ Paired t-test.

In all the specific health outcome measures for the diabetes (HbA1c), renal (urea level and urea-to-creatinine ratio) and surgery (number of dressings for healing wounds) specialties, the NC cohort performed better than the non-NC cohort. In particular, the corresponding patients in the NC cohort demonstrated a significantly larger reduction in mean HbA1c in six months than the non-NC cohort (mean reduction=-1.8% vs -0.2%, p<0.001, Table 3). Both urea level (mean=21.7 mmol/L vs 26.2 mmol/L, p=0.033, Table 3) and urea-to-creatinine ratio (mean=27.0 vs 34.8, p=0.004, Table 3) were better in the NC than the non-NC cohort just before discharge from the indexed hospital admission. The number of dressings for wound healing was significantly smaller in the NC than non-NC cohorts during the indexed hospital stay (median=7 vs 28, p<0.001, Table 3).

Patient satisfaction scores on the PSI are given in Table 4. Of particular note, the mean overall score of the NC cohort was 106.3 ± 9.0, given that the range of this score is 25 to 125 [16]. The mean PSI sub-scale scores of the NC cohort and the reference ranges of the mean PSI subscale and overall scores are also listed in Table 4.

**Table 4:** Patient satisfaction under NC care

	**Current study **^**1**^	**Previous published studies **^**2**^	
**Patient satisfaction instrument (PSI)**	Mean (SD)	Median (IQR) ^3^	Range ^3^
Professional care subscale score [range: 7 – 35]	31.3 (3.0)	29.6 (27.6 – 30.2)	(21.8 – 30.5)
Trust subscale score [range: 11 – 55]	46.7 (4.4)	40.2 (39.3 – 42.4)	(36.7 – 44.6)
Education subscale score [range: 7 – 35]	28.3 (2.9)	25.5 (24.5 – 26.0)	(20.3 – 28.8)
Overall satisfaction score [range: 25 – 125]	106.3 (9.0)	96.8 (93.3 – 98.2)	(84.8 – 103.5)

IQR: inter-quartile range.

^1^ The mean and standard deviation of the PSI scores of the current study.

^2^ Since there are no benchmark values of the PSI scores, an empirical approach has been adopted to generate reference ranges for the mean PSI subscale and overall scores. All the studies published in English during the period 1 Aug 2003 to 31 July 2013 and reporting PSI scores have been extracted through Google Scholar. A total of seven studies with 14 cohorts’ PSI scores reported were identified, please see the reference list [18-24].

^3^ The median (inter-quartile range) and range of the extracted studies’ reported mean PSI scores of the 14 cohorts are listed for reference.

## Discussion

This paper reports the findings of a quantitative study to explore the impact on selected patient health and service outcomes and satisfaction levels of introducing NCs to Hong Kong public hospitals, and is the quantitative part of a pilot study [14] to review and evaluate in a preliminary and independent way the first wave of seven new NC positions in Hong Kong’s public health care system in January 2009. With the successful establishment of the NC positions and their favourable outcomes, the Hospital Authority further expanded the NC positions to over 50 in 2012 [26].

To the best of our knowledge, this is the first study to examine the effect of introducing NCs to a public healthcare system in a Chinese society, and part of the limited evidence showing positive findings on patient health and service outcomes [10,27,28]. Our study used a historically matched controlled trial design, controlling for age, sex and disease type, to examine the impact of the NC programme on patient outcomes, and demonstrated that the NC cohort had favourable patient health and service outcomes in comparison with the non-NC cohort. Together with the high level of patient satisfaction, these findings indicate that the introduction of NC to Hong Kong public hospitals may affect the overall quality of patient care, of which patient outcomes are an essential component [29], in a positive way.

The qualitative part of our overall review and evaluation of the first wave of the NC programme in Hong Kong’s public hospitals [14] has identified five core functions of NCs in clinical environment: expert practice, leadership, education and training, practice and service development, and research [14], all of which are essentially consistent with those reported in pervious studies [12,30-32]. With these core functions, we suggest that the introduction of NCs in specialty units not only raises the overall nursing care standards but also boosts the morale and enthusiasm of the whole nursing team, which are the key factors motivating people to perform in an outstanding way [33]. Indeed, the present study highlights the significant implications of these NC core functions for patient health and service outcomes.

The present study results show that the proportion of patients in the NC cohort registering accident and emergency visits and hospital admissions in the first six months after being discharged from their indexed hospital admissions is significantly smaller than in the non-NC cohort. A closer look at the data reveals that those patients with further accident and emergency visits or hospital admissions were almost all from the chronic disease specialties (diabetes, renal and psychiatrics), except one or two patients coming from the continence specialty in either cohort (Table 3). This suggests that an NC in a chronic disease specialty may have a particularly promising role in reducing further accident and emergency visits and hospital admissions. Patient education is an indispensable component of chronic disease patient care, and one of the main roles of an NC [14,32,34,35]. In fact, proper and adequate patient education has been found to be associated with fewer hospital re-admissions in some chronic patient populations [36,37]. All these indicate that an NC may strengthen patient education and enhance patients’ self management of chronic disease.

More improvement in glycaemic control among the NC cohort, to a certain extent, may again be attributable to the strengthened patient education provided by NCs. The better performance in the patient health outcomes in the renal and surgical specialties and the number of acute complications during the hospital stays of the indexed admissions of the patients in the NC cohort may be associated with an increased exposure to expert clinical care from the NCs, who are not much involved in administration, the majority of their clinical time being spent directly on patients, while providing leadership in patient education, clinical teaching and professional consultancy to other healthcare professionals [14]. All the NCs are highly experienced and knowledgeable experts in patient care in their specialties, and it is expected that, with their direct care and supervision on frontline healthcare professionals, the overall quality of care can be enhanced, in turn improving patient outcomes. Besides a further improvement in direct patient care, NC’s professional leadership role can also boost the morale and enthusiasm of their nursing colleagues [33]. In fact, the significantly more frequent alterations to treatments or medication regimens on the part of nurses in the NC cohort indicates that the whole nursing team have more confidence in caring for patients, in which the professional leadership role of NCs may be a key factor.

In this study, we also found that patient satisfaction towards the care they received in the NC cohort was at a good level. Although constrained by the retrospective nature of the non-NC cohort, we were not able to make comparisons of satisfaction scores between the two cohorts. However, we observed remarkably high satisfaction scores (PSI) reported by the NC cohort compared with those reported by other cohorts in the literature (Table 4). These high PSI scores generally indicated that the NC cohort were happy with the care they received. Patient satisfaction is not only an integral part of high quality of care but is also associated with favourable patient outcomes [38,39], this may partly explain our findings.

There were several limitations to the study. First, owing to its retrospective nature and the limited data available, only unadjusted comparisons could be made in respect of patient outcomes; other factors not considered in the study, particularly the duration and severity of disease and length of the indexed hospital stay, might confound the comparison results, although the NC and non-NC cohorts were matched in age, sex and disease type. Furthermore, this was an observational study, and causal relationships between the introduction of NCs and favourable patient health and service outcomes could not be established. Second, there may be some selection bias in the study, although a prior subject selection algorithm to minimize such bias was used to select patients systematically into the study. Third, there might have been variations in the actual roles of the NCs played in their units, affecting the generalisabililty of the study findings. Forth, the internal validity of the satisfaction findings cannot be guaranteed without a comparison with a control group. The study was planned after the introduction of the NC programme. In addition to the selection of a control for comparisons, another major difficulty of the study was the choice of appropriate outcome measures sensitive to the impact of an NC. There is little in the literature providing insights in this regard [13,30]. Although the outcomes were chosen on the basis of appropriateness and feasibility [15], the validity of such outcomes in relation to the impact of NCs cannot be assured, and some caution is therefore needed in interpreting the findings of the study. In addition, no specific outcomes in the area of continence and psychiatric care were identified prior to data collection, leading to missing information on the impact of NC in these two specialties. Nevertheless, we believe that, despite the above limitations, the study may still provide useful insights for planning a large-scale patient outcome evaluation study.

## Conclusions

In summary, this study demonstrates that with the introduction of NCs in specialty units may have positive effects on patients’ health and service outcomes. The high satisfaction scores indicates good acceptability of the care provided to patients with the introduction of NCs, which is also reflected in the better patient outcome findings in the NC cohort. The preliminary patients’ health and service outcome findings support the introduction of NCs into the public healthcare system in Hong Kong. Further larger-scale studies are warranted to confirm the impact of NC positions on patient’s health and service outcomes.

## Competing interests

The authors declare that they have no competing interests.

## Authors’ contributions

DL, CC, SYC, SF and EC contributed to the conception and design of the study. DL, CC, SYC and DC prepared the proposal and supervised the study. DC supervised the data collection and takes responsibility for acquisition of data. DL and KCC have full access to all of the data in the study and take responsibility for the integrity of the data and the accuracy of the data analysis. KCC was responsible for data management and analysis. DL, KCC, CC and SYC interpreted the results and prepared the manuscript. DL, KCC, CC, SYC SF and EC contributed to critical revision of the manuscript. All authors read and approved the final manuscript.

## Pre-publication history

The pre-publication history for this paper can be accessed here:

http://www.biomedcentral.com/1472-6963/13/431/prepub
